# Activity of an anaerobic *Thermoanaerobacterales* hydrolase on aliphatic and aromatic polyesters

**DOI:** 10.3389/fbioe.2024.1520680

**Published:** 2025-01-17

**Authors:** Chiara Siracusa, Lisa Fohler, Lukas Leibetseder, Gerald Striedner, Chris Oostenbrink, Felice Quartinello, Georg M. Guebitz, Doris Ribitsch

**Affiliations:** ^1^ Department of Agrobiotechnology, Institute of Environmental Biotechnology, IFA-Tulln, BOKU University, Tulln an der Donau, Austria; ^2^ acib GmbH, Tulln an der Donau, Austria; ^3^ Institute of Bioprocess Science and Engineering, BOKU University, Vienna, Austria; ^4^ Department of Material Science and Process Engineering, Institute of Molecular Modeling and Simulation, BOKU University, Vienna, Austria

**Keywords:** enzyme-based recycling, polymer processing, biochemical characterization, aromatic polyesters, aliphatic polyesters

## Abstract

This study focuses on the biochemical characterization of a new hydrolase (Thb) expressed from anaerobic *Thermoanaerobacterales,* which could be used to improve biogas plant efficiency for plastic waste treatment. The specificity of Thb for various polyesters, including polyethylene terephthalate (PET), polybutylene adipate terephthalate (PBAT), polybutylene succinate (PBS), and polylactic acid (PLA), was compared to the well-studied cutinase HiC from *Humicola insolens*. Based on gravimetric analysis and quantification (high-performance liquid chromatography (HPLC)) of monomers solubilized upon enzymatic hydrolysis, Thb was found to be more active on aromatic polyesters, while HiC led to a higher amount of hydrolysis products on aliphatic polyesters (PBS and PLA). Polyester hydrolysis was further investigated by scanning electron microscopy and infrared spectroscopy. A comparison of the two enzyme structures indicated the higher aromatic character of specific regions of the Thb surface as a possible reason for these differences in specificity.

## Graphical Abstract

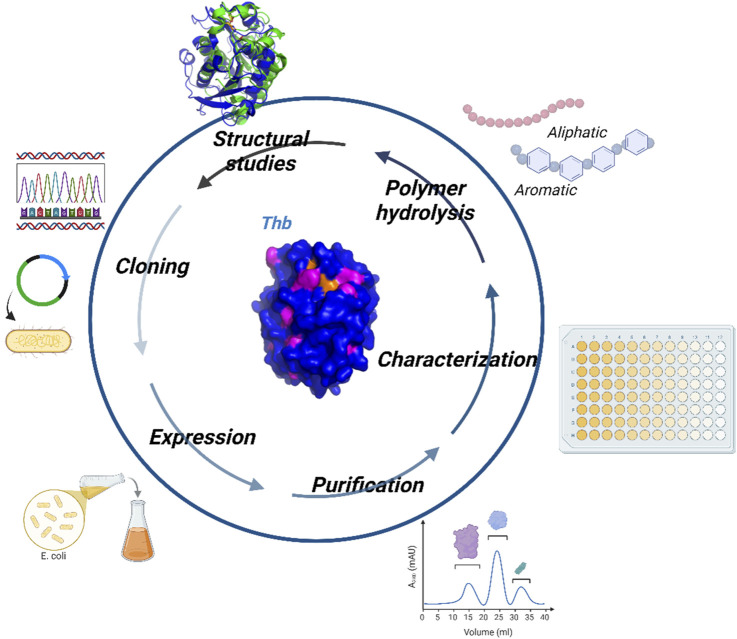

## 1 Introduction

Polyesters are widely used in food packaging materials due to many advantageous characteristics, such as barrier properties. Bio-based and/or biodegradable polyesters offer an environmentally friendly alternative to fossil-based recalcitrant polyethylene terephthalate (PET), and their market shares are expected to triple in the next few years in Europe (European bioplastics, 2023). The majority of food packaging products are meant to be single use for safety concerns, contributing to crowd waste streams, especially when contaminated with food residues. They are, therefore, preferentially treated in composting or biogas plants; unfortunately, they constitute up to one-fourth of the total plastic production (Ceballos-Santos et al., 2024). Yet, for example, PLA, the material with the highest production capacity among biobased alternatives to PET (31% of total bioplastics in 2023) (European bioplastics, 2023), is not home-compostable and requires thermophilic conditions (Narancic et al., 2018). Likewise, in biogas plants, biobased/biodegradable polyesters degrade well under thermophilic conditions but may require longer treatment times (Narancic et al., 2018).

As shown for other polymers like lignocellulose/hemicellulose, the addition of microorganisms specialized in decomposing these polymers to biogas plants (i.e., bioaugmentation) can improve their hydrolysis and enhance biogas yields as demonstrated in our group and other works (Weiß et al., 2016). Likewise, we have shown that the addition of anaerobic *Clostridium hathewayi*, which produces polyester-hydrolyzing enzymes, can boost the decomposition of polyesters from packaging materials (e.g., PBAT) in biogas reactors (Perz et al., 2016a). Thermophilic microorganisms with these capabilities would be beneficial because many biogas plants operate at thermophilic conditions, and polyesters are hydrolyzed faster at higher temperatures (close to their glass transition temperature).

In the past, thermophilic mixed cellulolytic consortia were enriched from an industrial-scale biogas fermenter (Strang et al., 2017). Next-generation sequencing of the consortia revealed that the main orders are *Thermoanaerobacterales* and *Clostridiales*. Within the consortia, the predominant strains were *Thermoanaerobacterium thermosaccharolyticum, Caldanaerobacter subterraneus, Thermoanaerobacter pseudethanolicus*, and *Clostridium cellulolyticum*. Bioaugmentation experiments showed that the consortia enhanced the methane yield from corn stover by 22%–24%. Strains from these phylogenetic orders might, therefore, also improve the degradation of polyesters. A previous study has shown that *Clostridia*, more specifically *C. hathewayi*, can degrade PBAT, mostly applied in flexible food packaging bags (Perz et al., 2016a). The activity of polymers is based on the presence of enzymes that these microbes produce and usually secrete. For example, an esterase was isolated from the above-mentioned *C. hathewayi* that hydrolyzes the synthetic compostable polyester PBAT. Two further hydrolases with PBAT cleavage capacity were identified from *Clostridium botulinum* (Perz et al., 2016b). Such polyester-hydrolyzing enzymes could also have a potential for recovery of polyester building blocks to be then separately collected (e.g., from blended textiles or multilayer materials) (Pellis et al., 2023).

In recent years, remarkable progress has been made in exploiting novel biotechnological technologies, ranging from metagenome screening to enzyme engineering, in order to make enzymatic recycling of “recalcitrant” PET industrially feasible (Biundo et al., 2018; Sonnendecker et al., 2022; Arnal et al., 2023). Cutinases from *Humicola insolens*, *Thermobifida cellulosilytica*, or *Thermobifida fusca* showed enhanced depolymerization activity from 55°C to 70°C and against moderately crystalline PET substrates (Romano et al., 2023). Conversely, *ls*PETase from *Ideonella sakaiensis* (Yang et al., 2016) retains the ability to catalyze PET hydrolysis at milder temperatures. The leaf and branch compost (LCC) and PHL7 cutinases, which both originated from the metagenomic screening of compost, outperformed the previously mentioned ones, at least for PET hydrolysis in the range 65°C–70°C, close to the PET glass transition temperature (Tg). Yet, there is still a need to expand our knowledge about polyester hydrolyzing enzymes from microorganisms originating from different ecosystems.

Therefore, to better understand the fate of different polyesters from packaging waste under anaerobic conditions, we have investigated a polyester hydrolyzing enzyme from an anaerobic zone of a thermophilic composting plant (Martins et al., 2013). The enzyme, named Thb, originates from a member of the anaerobic *Thermoanaerobacterales*, which can grow at up to 80°C and was characterized in terms of its ideal reaction conditions (temperature, buffer, and pH). The specificity of this enzyme was investigated on aliphatic and aromatic polyesters and compared to the well-characterized *H. insolens* cutinase. Therefore, the hydrolysis of PET of the aliphatic polyesters PLA and PBS and of PBAT as aliphatic/aromatic copolyester was compared. The results should both be valuable as a basis for the development or modification (Arnal et al., 2023) of new polyester active enzymes as well as for bioaugmentation strategies in anaerobic processes.

## 2 Materials and methods

### 2.1 Chemicals and polymers

Buffer components (K_2_HPO_4_ and KH_2_PO_4_, Trizma base, and NaCl), bovine serum albumin (BSA), and *p*-nitrophenol butyrate (*p*-NPB) were purchased from Sigma-Aldrich (USA). Amorphous polyethylene terephthalate (PET) and crystalline PET (crystallinity >50%, size<300 µm) were purchased from Goodfellow. Polybutylene adipate terephthalate (PBAT) was purchased from BASF, and polylactic acid (PLA) and mixed waste containing PET (MW) were provided by AIMPLAS. Lysogeny broth (LB), kanamycin, and isopropyl-β-D-thiogalactoside (IPTG) were purchased from Sigma-Aldrich (Germany), while *Escherichia coli* BL21-Gold (DE3) competent cells were purchased from Agilent.

Chemicals for fermentation media and semisynthetic medium were purchased from Sigma-Aldrich (Germany) where not otherwise specified.

### 2.2 Polymer preparation

Polyester films were sized to 0.5 cm × 1 cm, washed in Triton X-100 (5 g/L) and sodium carbonate (100 mM), and rinsed with ultrapure water. Additionally, the same polymers were cryomilled with two cycles of 1:30 min each with a frequency of 30 s^−1^, with a 1 min at 5 s^−1^ cooling step in between. Powders were afterward homogenized with a sieve of 500 µm pore size. Crystalline PET was already available as a powder with <300 µm size.

### 2.3 Expression and purification of the hydrolase Thb from *Thermoanaerobacterales*

#### 2.3.1 Expression of Thb in *Escherichia coli* and in working cell bank

A hydrolase from *Thermoanaerobacterales* (Thb, NCBI accession number MBO2503201.1) was codon-optimized for *E. coli* and cloned with an N-terminal Strep-tag over the restriction sites NdeI and HindIII into vector pET26b (+), yielding pET26b (+)_Strep_Thb, and expressed in competent *E. coli* BL21-Gold (DE3) as previously reported (Biundo et al., 2016). A first 24-h pre-culture in LB medium (total volume 10 mL LB) supplemented with kanamycin (40 μg/mL) as a selection marker was followed by the preparation of a main culture with a starting OD_600_ of 0.1. After 16-h growth at 37°C, 150 rpm in Erlenmeyer flasks (total volume 200 mL LB), the clones were induced with 0.08 mM IPTG and allowed to grow at a lower temperature (20°C) while shaking at 150 rpm. The harvesting of the cells was done by aliquoting the total 200 mL cell culture into 50-mL Falcon tubes, which were centrifuged at 3,700 rpm, 4°C, 20 min.

For the larger volume expression, 2 mL of working cell bank (WCB) of the employed production strain (enGenes e^X^-press V2 with pET26b (+)_Strep_Thb plasmid) were added to 30 mL of semi-synthetic medium (SSM) (Supplementary Table S1, ESI) containing 50 μg/mL kanamycin in a 500-mL baffled boom flask. The pre-culture was incubated for 5 h at 37°C and 200 rpm. For the inoculation of 500 mL batch medium (composition see Supplementary Tables S2, S3, ESI) in the 2-L benchtop bioreactors (DASGIP Bioblock, Eppendorf SE, Hamburg, Germany), the OD_600_ of the pre-culture was measured, and an amount of cell suspension equaling 25 OD_600_ units was used. The temperature was kept at 37 (±0.2)°C. The pH control was set to 7.0 (±0.1) and regulated by dosing 12.5% ammonia solution. The dissolved oxygen (DO) cascade was designed to maintain the DO at 30% by first accelerating the stirrer speed (400–1,200 rpm), then increasing the air flow rate (10–200 sL/h), and finally increasing the oxygen concentration in the gas flow (21%–100%). Foaming in the reactor was kept under control by dosing antifoam agent (1:10 diluted, Struktol J673A, Schill + Seilacher, Böblingen, Germany) when needed. The first feed was started immediately after the batch phase ended with a cell dry weight (CDW) of 10 g/L reached in 8.25 h. Two feed phases were employed. First, an exponential feed phase (µ = 0.135 h^−1^) was used to reach a biomass of 40 g/L of CDW in the bioreactor. Subsequently, a linear feed profile was set (fixed feed rate of 0.12 g glucose/min, 30°C, and pH 6.5). With the start of the linear feed, the growth of the host cells was halted by the addition of 10 mM arabinose to express the T7 phage protein Gp2, thereby decoupling growth and recombinant protein production. In parallel, the enzyme expression was induced with 1.2 µmol IPTG/g CDW. The first sample was taken right before induction as a reference and to determine possible basal expression of Thb. Further samples were drawn every 2 hours for a maximum of 10 hours. After 10 h, the fermentation broth was harvested by separating the cells from the medium via centrifugation (15,400 g for 1 h at 10°C). The biomass and supernatant were frozen separately at −20°C until further processing.

#### 2.3.2 Purification of expression-derived biomass for Thb isolation

The pellet was then recovered, weighed, and resuspended in buffer W (100 mM Tris HCl, 150 mM NaCl, pH 8) in a 5 mL per 1 g ratio. The mixture was sonicated in an ice cooling environment in 10 cycles each of 10 s, with 2 min interruptions in between each cycle (SONICATOR BRANSON Ultrasonics cell disruptor (USA)). The sonicated material was removed from cell fragments and debris via centrifugation (12,000 rpm. 4°C, 20 min) and further filtered with a PES filter (pore size 0.2 µm, Nalgene) before being loaded onto a column for affinity chromatography in the AEKTA purification system, as the enzyme was provided with a Strep-tag. The elution was done through the addition of buffer W, which was implemented with biotin. The affinity chromatography column (Strep-Tactin^®^ XT Sepharose, volume 5 mL, Cytiva) in use was provided with a Strep-tag affine matrix to capture the Strep-tagged enzymes. The eluted fractions were pooled and concentrated through an ultrafiltration filter (Amicon, 10 kDa cutoff Merck, Germany). Before storage at −20°C, the buffer was changed into 100 mM potassium phosphate (KPO) pH 8.0 by the use of PD-10 desalting columns (PD-10 desalting column, Cytiva). The cutinase from *H. insolens* (HiC) was from Novozymes and used without any further treatment. The efficiency of purification was tested through an activity assay using *p-*NPB as substrate and by SDS-Page. Load, flowthrough, and eluted fractions were diluted in Laemmli buffer with 2-mercaptoethanol (Information, 2000) and loaded onto pre-cast gels (Mini-PROTEAN TGX Stain-Free, Bio-Rad), run at 150 V for 30 min. The protein marker IV (Peqlab, Germany) was the reference for the molecular weight bands.

### 2.4 Protein quantification, activity, and temperature stability

The protein concentrations of both the HiC and eluted Thb fractions were estimated through a Bradford-based Bio-Rad Protein Assay (Bio-Rad Laboratories GmbH, Munich, Germany) using bovine serum albumin (BSA) as the standard. The activity of HiC was measured in the final buffer used for polyester hydrolysis (1M KPO), while Thb activity was characterized in different buffers and molarities. The substrate for all the assays was *p*-nitrophenyl butyrate (*p-*NPB), and the volumes in use were kept constant for all the samples: the enzyme was diluted accordingly into the buffer of analysis (at set pH and molarity). The volume of each sample was 20 μL, placed in triplicate in a 96-well plate. A 200-μL aliquot of *p-*NPB containing solution (obtained diluting the substrate in the buffer of interest) was added to the samples and to a triplicate set of blanks (buffer only), and the increase of absorbance was recorded for 5 min at 405 nm on a plate reader (Tecan infinite M200, Tecan Austria GmbH). The same measurement was repeated after dilution in pre-heated buffers (ranging from 50°C to 60°C–70°C). The results were expressed in units (U) per mL, where U corresponds to the amount of enzyme that catalyzes 1 µmol of substrate per minute. Thermostability was also evaluated through activity assessment for 72 h (with time points at 0 h, 6 h, 24 h, 48 h, and 72 h) to evaluate the loss of activity in a real incubation set-up, in the buffers in which the activity was measured as the highest. The values obtained through the *p-*NPB assay after each time point were measured and compared to the assessed initial one (indicated as 0 h) and assumed as 100%. These measurements were performed on enzyme fractions incubated at 30°C, 50°C, and 70°C.

### 2.5 Enzymatic hydrolysis of polyesters

Polyester powders were incubated in 2-mL Eppendorf tubes with a total reaction volume of 2 mL, where Thb or HiC was diluted to 1 µM final concentration. The films were sized to be 1 cm × 0.5 cm, while 10 mg of each powdered polymer was incubated in the same buffer volume. The buffer in use was 1 M KPO pH 8. The Eppendorf tubes were placed horizontally in an orbital shaker incubator at 70°C and 150 rpm shaking. Hydrolysis of the powder polymers was performed also with Phl7, expressed and produced in *E. coli* as described in (Fohler et al., 2024).

### 2.6 Analysis of weight loss and released monomers

After incubation of the polyester samples, they were washed in the three subsequent steps as summarized for the preparation prior to hydrolysis to remove any possible attached residuals. The polymers were dried and weighed to compare with the original weight and to calculate their mass loss, which was reported as a percentage. The released monomers were quantified by using high-performance liquid chromatography (HPLC) in two different settings. An Agilent Technologies 1260 Infinity instrument was used in combination with a C18 reversed-phase column. Terephthalic acid (TPA) was detected via a UV lamp, and the method used was based on a gradient of methanol and 0.1% formic acid (flow 0.85 mL/min), with an injection volume of 10 µL. The samples were diluted in methanol and acidified with 30 µL 6 N HCl before being centrifuged at 4°C (15 min and 14,000 rpm) and filtered through 0.2-µm polyamide filters into HPLC vials. All other monomers were detected through their refractive index (using a Transgenomic IC SEP-ION-300 and a mobile phase of 0.01 N H_2_SO_4_, with a flow rate of 0.325 mL/min, 45°C). The Carrez protocol was used to remove proteins through the addition of potassium hexacyanoferrate (II) trihydrate and zinc sulfate heptahydrate reagents. The samples were centrifuged for 30 min at 4°C and 14,000 rpm before filtering into HPLC vials through 0.2-µm polyamide filters. Calibration curves were prepared with serial dilution of all monomers (individually either in buffer or ultrapure water according to each monomer detection protocol) and used to estimate their concentrations in the hydrolysates.

### 2.7 Model structure comparison

A homology model of Thb was created with the Swiss model server (Waterhouse et al., 2018) in the automated mode. The crystal structure of PHL7, entry 8BRA in the protein databank, with a sequence identity of 90%, was used as a template; see Supplementary Figure S1 in the supporting material for the sequence alignment. The obtained model showed a QMEANDisco score of 0.87 (Studer et al., 2020). An Alphafold 3 (Abramson et al., 2024) model for Thb was also reported. The resulting model was compared to the crystal structure of HiC, with PDB code 4OYY, by aligning the catalytic triads of both models.

## 3 Results

The large variety of available metagenomic databases can facilitate the identification of new microbial enzymes in specific environments, reducing the strict need for complex functional screenings. Such *in silico* screening based on already known polyester hydrolases was applied in this study to identify a new thermostable polymer hydrolase in an anaerobic consortium. In the course of the screening, starting with an “anaerobic” polyester hydrolyzing enzyme that we had previously identified in *C. hathewayi*, an alpha/beta hydrolase from the *Thermoanaerobacterales* bacterium (accession number MBO2503201.1) was identified in a compost (anaerobic zone) metagenome (Martins et al., 2013) and named Thb. Hydrolase Thb has only low homologies to the hydrolase from *C. hathewayi* (18%), the esterase A from *C. botulinum* (25%), and the *Humicola insulens* cutinase (23%). In comparison, the homologies to IsPETase (60%), LCC (65%), and PHL7 (90%) are significantly higher. The enzyme was produced in *E. coli* and characterized, and the specificities were compared with HiC.

### 3.1 Expression of Thb in *Escherichia coli* and purification

Affinity-based purification by an N-terminal Strep-tag yielded a pure protein with a molecular weight of 35 kDa according to SDS-PAGE analysis (Supplementary Figure S2 (ESI)). While the small-scale culture (total volume 200 mL in shake flasks) gave 2.64 mg_enzyme_/L_culture_ or alternatively 0.27 mg_enzyme_/g_biomass_, this yield was significantly improved when the culture was upscaled to the 2 L bioreactor with overall better control of the parameters and optimized media component (Supplementary Tables S1–S3). The final purified enzyme was, in fact, 33 mg_enzyme_/L_culture_ or 0.84 mg_enzyme_/g_biomass_ from the bioreactor cell biomass, proving that the upscale was not only possible but also allowed higher final yields.

The specific activity on *p*-NPB as a substrate (1 M KPO buffer, pH 8, T 70°C) was 54.1 U/mg. The activity based on the same model substrate (*p*-NPB) was also compared in various incubation conditions regarding buffer type (Tris HCl and KPO), molarity, temperature, and pH. The most suitable environment proved to be KPO, for which the highest activity of 152.5 U/mg was found on 100 mM KPO pH 8 and 60°C (Supplementary Figure S3, ESI). The better results for KPO than Tris HCl confirmed the previous finding of a putative inhibitory effect of Tris HCl on enzyme activity in favor of the adoption of KPO, whose phosphate ions were expected to stabilize the enzymes (Schmidt et al., 2016; Gamerith et al., 2017; Weinberger et al., 2017).

Thermostability at specific temperatures and conditions was tested in the next step. In agreement with previous results (Daniel and Danson, 2013; Schmidt et al., 2016; Ismail et al., 2024), enhanced stability was seen at the highest KPO molarity of 1M (50°C: 10.8 U/mg; 60°C: 48.7 U/mg; 70°C: 54.1 U/mg), while a reported large stabilization effect of Ca^2+^ (Sonnendecker et al., 2022) could not be confirmed when CaCl_2_ was added to a final concentration of 2 mM.

In addition, the *p-*NPB assay was also carried out under the incubation conditions used for polymer hydrolysis (1 µM concentration in the presence of PET stripe (1 cm × 0.5 cm) with time points at 6 h, 24 h, 48 h, and 72 h (Supplementary Figure S4A). Additionally, 30°C and 50°C were considered to monitor the effect of temperature on the long-term stability (Supplementary Figures S4B, 4C). Thb and HiC followed a similar trend regarding stability. After 6 h of reaction, the activity on *p-*NPB decreased by more than 50%, while at the end of the incubation (72 h), the residual activity was 20% for Thb and <5% for HiC. Lower temperatures also caused significant activity loss (Supplementary Figures S4B, 4C). In particular, while the stability of HiC at 50°C was retained >10% for the whole experiment (owing to various additives and stabilizers in the commercial formulation), Thb at the same temperature was barely active at 48 h, maintaining a low basal activity until 72 h when compared with the initial one. These results confirmed the destabilization of the enzyme in buffer at high temperature for longer incubation time, which is a common response of enzymes to heat. However, a sharp decrease in the residual activity in the reaction mixture may also indicate partial adsorption of the enzyme on the present PET. Moreover, it must be taken into account that the activity assay on *p*-NPB was carried out on an aliquot of the soluble fraction of the enzyme, which only estimated the actual available free enzyme and could be affected by several parameters throughout the duration of the incubation. Therefore, in the next step, the activity of the polymer was assessed *via* weight loss and released monomer.

### 3.2 Hydrolysis of different aromatic and aliphatic polyesters

The specificity of Thb on a range of aromatic or aliphatic polyesters was compared to the *H. insolens* cutinase, which has been well studied for polyester hydrolysis. Previously (Marten et al., 2005; Gigli et al., 2019), a higher susceptibility to enzymatic hydrolysis was reported for aliphatic polyesters than the aromatic polyester. Weight loss and soluble molecules released from the polyesters were quantified to assess whether Thb followed this hypothesis.

#### 3.2.1 Weight loss and surface changes of polyester films

The polymer films recovered after the hydrolysis were washed as in the preparation step. PET, PBAT, and PLA were hydrolyzed to different extents by Thb and HiC. PET was completely hydrolyzed by Thb after 48 h (Supplementary Figure S5), while a maximum weight loss of only 38% was seen for HiC after the same incubation time. In agreement with these data, higher hydrolysis rates were previously reported for the PHL7, which is closely related (90%) to Thb (Sonnendecker et al., 2022) when compared to HiC. In contrast, a slightly faster decomposition of PLA was seen for HiC when compared to Thb; its mass was reduced by approx. 25% vs. 17% after 72 h of incubation. Likewise, the aliphatic/aromatic polyester PBAT was decomposed faster by HiC, even though both enzymes led to complete decomposition after 24 h. For crystalline PET, only a marginal weight loss was seen for both enzymes (data not shown).

**FIGURE 1:**
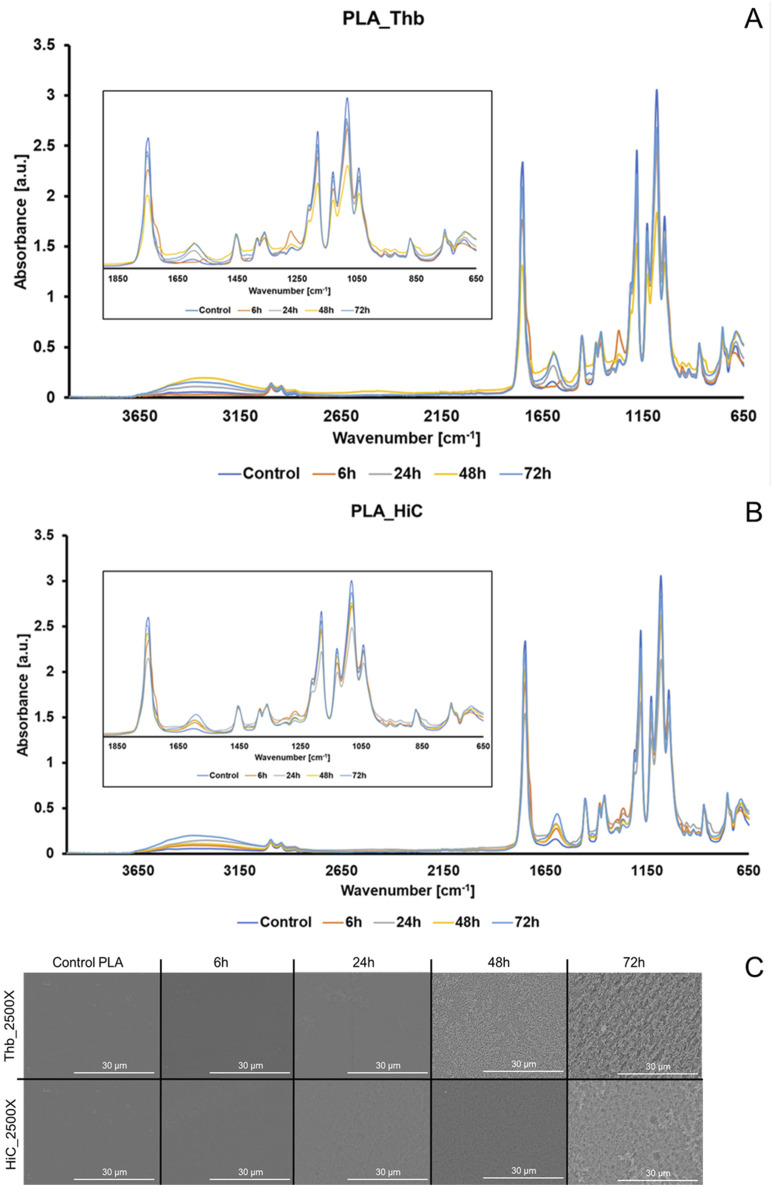
FT-IR spectra with zoom between 1850 cm^−1^ and 650 cm^−1^ and SEM pictures for PLA residual films from hydrolysis. **(A)** FT-IR spectra of PLA treated with Thb; **(B)** FT-IR spectra of PLA treated with HiC (blue line: control, orange line: 6 h, gray line: 24 h); **(C)** SEM micrographs for PLA treated with Thb or HiC at ×2,500 magnification.

FT-IR spectra and SEM micrographs were acquired for the residual polymers. As shown in Figure 1, PLA-superimposed spectra for both enzyme-catalyzed reactions showed clear changes in peaks related to ester and free carboxylic group, namely, an increase in 1,620 cm^−1^ peak intensity (formation of free carbonyl groups from ester bond hydrolysis). Under SEM, PLA, and PBAT showed different patterns of surface erosion, especially PBAT, where the Thb-treated samples had deeper but less densely distributed cavities (Figure 1; Supplementary Figures S6–S8). Micrographs of PLA displayed instead an enhanced bulk decomposition with a homogeneous sponge-like structure after 48 h (Figure 3C). Regarding PLA hydrolyzed by HiC, a more dispersed pattern with deeper pores was seen after 72 h, which can be associated with locations of degradation (Figure 3C). Even though the aspect of the specimen at the SEM would indicate a more pronounced decomposition by Thb, according to weight loss and HPLC results, HiC was more active on PLA. Indeed, we have previously shown that on the same polyester, different enzymes may prefer acting “horizontally” on the surface or “vertically,” leading to crater-like structures (Zumstein et al., 2017). Similar results were recorded in the reactions conducted in the presence of 2 mM CaCl_2_ (Supplementary Figure S7), suggesting the poor influence of a metal-dependent stabilizer effect in the applied settings.

#### 3.2.2 Released monomers

**FIGURE 2:**
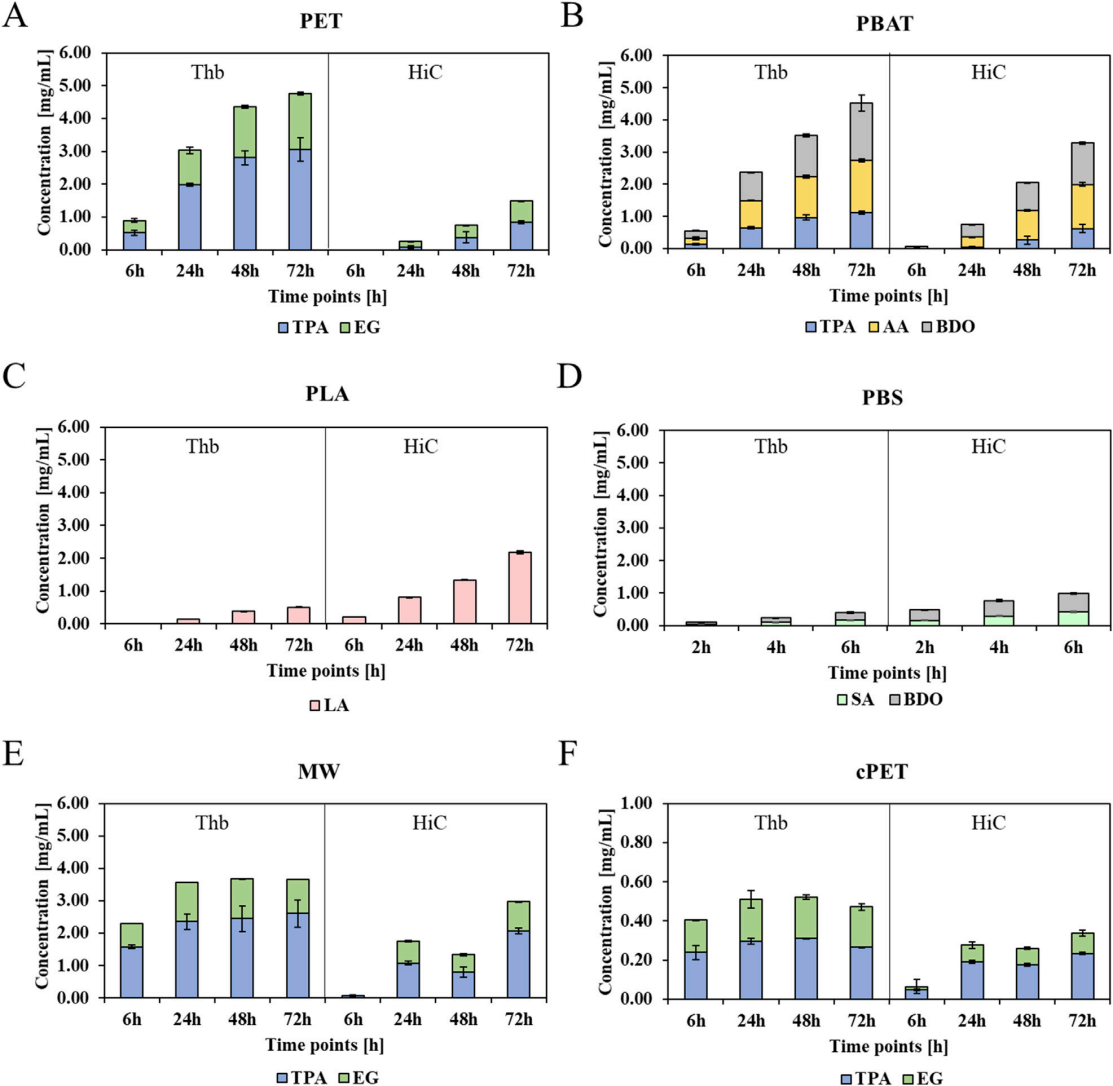
Soluble monomers released from PET, PBAT, PLA, PBS, mixed waste, and crystalline PET hydrolysate by the enzymes Thb and HiC. Values were normalized by the initial weight of the polymer in mg. Blue bars: TPA; green bars: ethylene glycol (EG); pink bars: lactic acid (LA); light green bars: SA; gray bars: 1,4-butanediol (BDO); yellow bars: adipic acid (AA). **(A)** PET, **(B)** PBAT, **(C)** PLA, **(D)** PBS, **(E)** MW, and **(F)** crystalline PET.

In agreement with the weight loss measurements, more than three times higher amounts of terephthalic acid (TPA) were released from the aromatic polyester PET by Thb than by HiC (Figure 2). Similar results were obtained for the hydrolysis of films (examples of chromatograms are available in Supplementary Figure S9, and plotted histograms for films can be seen in Supplementary Figure S10). Again, Thb caused a 3-fold higher released TPA from the PET film, while it was outperformed by HiC by more than 2-fold in the case of PLA hydrolysis. As expected, the aliphatic polyesters PLA and PBS were hydrolyzed significantly faster by HiC. For example, 0.4 mg/mL lactic acid (LA) hydrolyzed from PLA by HiC compared to only 0.2 mg/mL LA hydrolyzed by Thb were released after 72 h of incubation. In general, PBS was hydrolyzed faster by HiC than by Thb, leading to 0.4 mg/mL succinic acid (SA) after 6 h of incubation versus 0.1 mg/mL SA seen for Thb. The polyester PBAT containing both aromatic and aliphatic bonds was hydrolyzed better by Thb because almost double the amount of TPA was found in soluble form in the Thb hydrolysate after 72 h. This trend was confirmed by incubation at lower concentrations of the enzyme (Supplementary Figure S11, ESI).

Interestingly, both enzymes were also able to release monomers from mixed waste primarily containing PET: as expected, a higher amount was released from a Thb-catalyzed reaction (3.4 mg/mL TPA after 72 h) than HiC (1.6 mg/mL TPA), while both enzymes were generally slightly more active on the PET fraction of the mixed waste than on the PET standard. Finally, roughly ten times less monomers were released by the enzymes from crystalline PET, confirming the reported recalcitrance of crystalline regions toward enzymatic hydrolysis (Hwang et al., 2022). Yet, in agreement with the above results, Thb was more active on crystalline PET than HiC, with 0.4 mg/mL TPA traced in the hydrolysate after 72 h (0.2 mg/mL TPA after 72 h from HiC).

Although a proportional relationship between weight loss and released monomers was expected, enzymatic hydrolysis may first release soluble oligomers (which were not quantified here), which are afterward exo-wisely cleaved into monomers (Gigli et al., 2019). This may explain why, for PBAT, a higher weight loss was seen for HiC while a higher number of monomers was released by Thb. Yet, for PET and PLA, the release of monomers correlated with the weight losses. The incubation with 2 mM CaCl_2_ (HiC and Thb kept at the same concentration of 1 µM) resulted in no differences in terms of released monomers, confirming the weight loss findings. Reactions performed at 30°C are reported in Supplementary Figure S12, with a two-fold up to almost ten-fold decrease in hydrolytic efficiency when the amounts of released monomers are measured.

To place the study in the context with other polyester hydrolyzing enzymes, the activity of Thb was compared to Phl7 (Sonnendecker et al., 2022), which is the most closely related (90%) of the recently developed enzymes. When incubated at identical conditions (1M KPO, pH 8, 70°C), Phl7 was slightly more active on PET than Thb but less active on PBAT, PLA, and PBS (Supplementary Figure S13). The activity of Phl7 has previously been compared to other recently developed enzymes, such as LCC, which caused about 30% less weight loss of PET under the same conditions (https://doi.org/10.1002/cssc.202101062).

Increasing amounts of the different polyesters were incubated with equal concentrations of the enzymes. A constant enzyme amount with higher amounts of polyesters led to a higher monomer release, except for PBAT, where a plateau was reached (Supplementary Figure S14).

### 3.3 Modeling Thb and HiC

**FIGURE 3:**
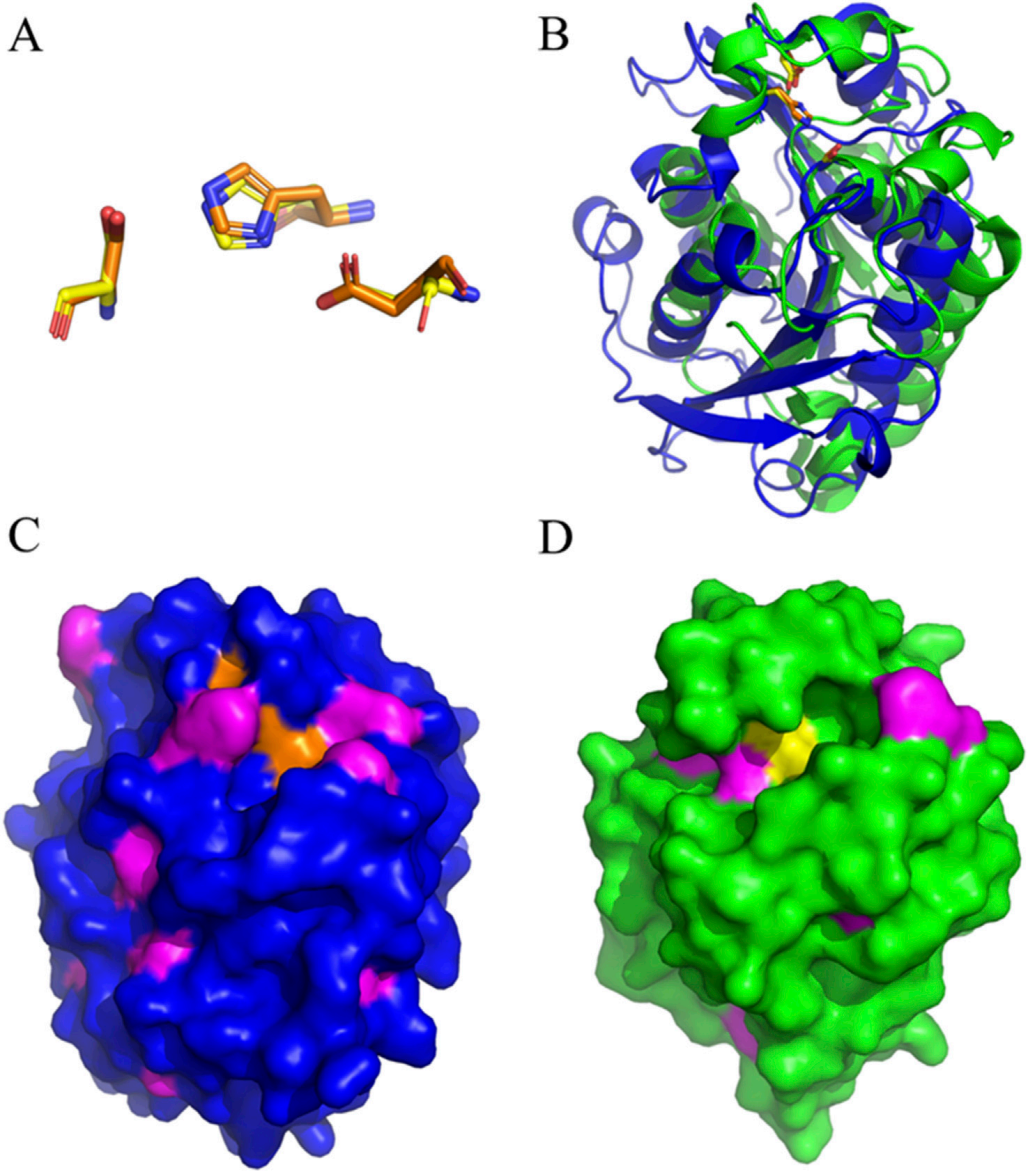
**(A)** Overlay of the Ser-His-Asp catalytic triad of Thb (carbon atoms in orange) and HiC (carbon atoms in yellow). **(B)** Overall comparison of the structures of Thb (blue) and HiC (green). **(C)** Surface representation of Thb with the catalytic triad in orange and aromatic residues in magenta. **(D)** Surface representation of HiC with the catalytic triad in yellow and aromatic residues in magenta. Yellow: catalytic triads; magenta: aromatic groups (Phe, Tyr, or Trp).

The overlay of the HiC structure with the one from Thb displayed significant differences between the two enzymes, offering a structural interpretation of their different specificity on aromatic and aliphatic polyesters. Strikingly, the HiC catalytic triad (Ser105, Asp160, and His173) aligned almost perfectly with that from Thb (Figure 3A). The Thb ɑ/β-hydrolase fold, in which the catalytic triad Ser131-His208-Asp177 is located, is conserved in other PET hydrolyzing enzymes. S131 provides the nucleophile site, while the acid D177 stabilizes the base H208 (Han et al., 2017) (Figure 3B). The Alphafold 3 model was virtually identical to the homology model, giving a root-mean-square deviation after an alignment of 0.16 Å (Supplementary Figure S15).

Serine hydrolases generally possess a cleft that determines substrate specificity. A surface representation is displayed in Figures 3C, D, where aromatic groups (Phe, Tyr, or Trp) are shown in magenta. Thb (Figure 3C) displays a shallow but wide cleft with a larger number of aromatic groups than HiC (Figure 3D). A wide and shallow but aromatic cleft can explain the preference for aromatic substrates of Thb, while the more pronounced cleft of HiC could preferentially accommodate less bulky, aliphatic substrates.

The co-crystallization of PHL7 with TPA was already carried out and demonstrates the interaction between the enzyme active site and its substrate. I179, W156, D177, H209, A131, F63, and M132 are responsible for the direct interaction of the enzyme with TPA (Richter et al., 2023). These are well-conserved in the Thb structure; therefore, the hydrolytic activity is not disrupted by the slight divergence between the two homologous proteins.

## 4 Conclusion

A new polyester hydrolyzing enzyme from *Thermoanaerobacterales* (Thb) derived from the metagenome from an anaerobic zone of a thermophilic compost plant was heterologously expressed and purified. Interestingly, Thb shows a clear preference for aromatic polyesters based on weight loss measurements and on the amount of monomers released when compared to the well-studied cutinase HiC. Combined with information obtained from structure/function analysis, this knowledge should facilitate future enzyme engineering towards specificity on desired polyesters. Moreover, thermophilic anaerobic microorganisms secreting polyester hydrolyzing enzymes could be used for bioaugmentation in biogas plants towards enhanced decomposition of polyester-based packaging materials.

## Data Availability

The datasets presented in this study can be found in online repositories. The names of the repository/repositories and accession number(s) can be found below: https://www.ncbi.nlm.nih.gov/, MBO2503201.1.
