# Supplementary material for: Activity of an anaerobic Thermoanaerobacterales hydrolase on aliphatic and aromatic polyesters
Source: Front Bioeng Biotechnol. 2025 Jan 17;12:1520680. doi: 10.3389/fbioe.2024.1520680 (PMC11782994; doi:10.3389/fbioe.2024.1520680)
Supplement: Supplementary file 1 [file Table1.DOCX]

***Supplementary Material***

**Activity of an anaerobic *Thermoanaerobacterales* hydrolase on aliphatic and aromatic polyester**

**Table S1. Semi-synthetic medium composition with relative final concentration**

| Order of addition | Media component | Amount feed |  |
| --- | --- | --- | --- |
| 1 | | KH_2_PO_4_ | 3,00 g/l |
|  |  | K_2_HPO_4_ | 4,58 g/l |
| 2 | | Na_3_Citrate dihydrate | 0,25 g/g CDW |
|  |  | MgSO_4_.7H_2_O | 0,10 g/g CDW |
| 3 | | CaCl_2_.2H_2_O | 0,01 g/g CDW |
| 4 | | Trace element solution | 50,00 µl/g CDW |
| 5 | | (NH_4_)_2_SO_4_ | 0,45 g/g CDW |
|  |  | NH_4_Cl | 0,37 g/g CDW |
| 6 | | Glucose Monohydrate | 3,30 g/g CDW |

**Table S2. Fermentation media composition with relative final concentration**

| Order of addition | Media component | Amount Batch | Amount Feed |
| --- | --- | --- | --- |
| 1 | KH_2_PO_4_ | 2.1 g/L | 8.3 g/L |
| 1 | 85% H_3_PO_4_ | 0.7 g/L | 2.8 g/L |
| 2 | Yeast extract | 1.5 g/L | --- |
| 3 | Na-citrate | 0.4 g/L | 4.1 g/L |
| 4 | MgCl_2_*6H_2_O | 0.5 g/L | 4.6 g/L |
| 5 | CaCl_2_*2H_2_O | 0.2 g/L | 2.0 g/L |
| 6 | Trace element solution | 500 µl | 5 mL |
| 7 | (NH_4_)_2_SO_4_ | 5.0 g/L | --- |
| 8 | Glucose | 28.6 g/L | 285.7 g/L |

**Table S3. Trace Element Solution composition with relative final concentration**

| Order of addition | Media component | Amount feed |  |
| --- | --- | --- | --- |
| 1 | | FeSO_4_^.^7H_2_O | 40,0 g/L |
| 2 | | MnSO4 ^.^H2O | 10,0 g/L |
| 3 | | AlCl3^.^6H2O | 10,0 g/L |
| 4 | | CoCl2 6H2O | 7,3 g/L |
| 5 | | ZnSO4^.^7H2O | 2,0 g/L |
| 6 | | Na_2_MoO_4_ 2H2O | 2,0 g/L |
| 7 | | CuCl_2_.2H2O | 1,0 g/L |
| 8 | | H3BO3 | 0,5 g/L |


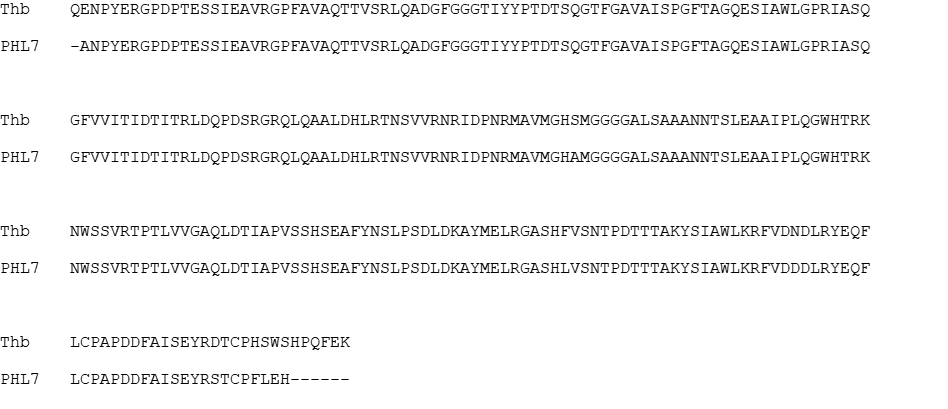


**Figure S1**: sequence alignment of Thb and PHL7, as used by Swiss Model.

**
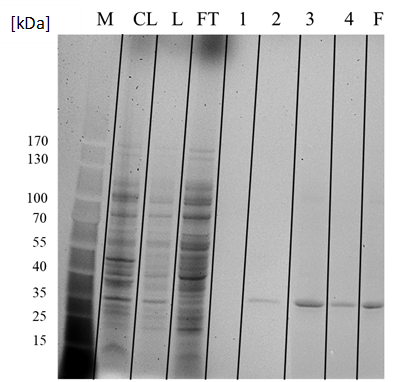
**

**Figure S2.** SDS Page analysis of the different fractions from purification. M: marker (Peq Gold IV), CL: cell lysate, L:load diluted 1:10, FT: flowthrough, 1,2,3,4: elution fractions, F: final fraction after collection and buffer exchange.


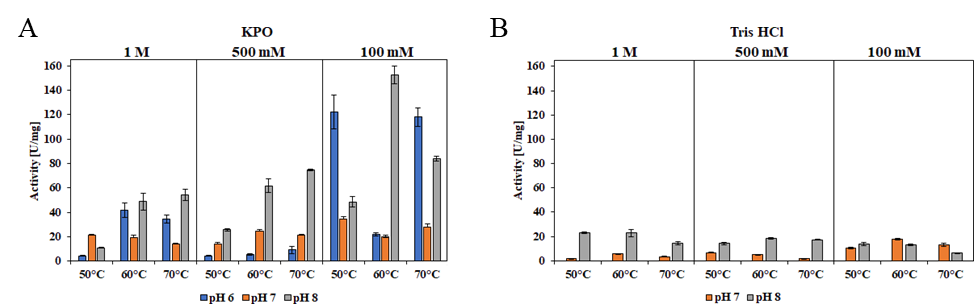


**Figure S3.** Comparison of activity (U/mg) measured on *p*NPB for Thb in the buffers KPO and TrisHCl at different pH, molarities and temperatures. A: KPO buffer, B: TrisHCl buffer.


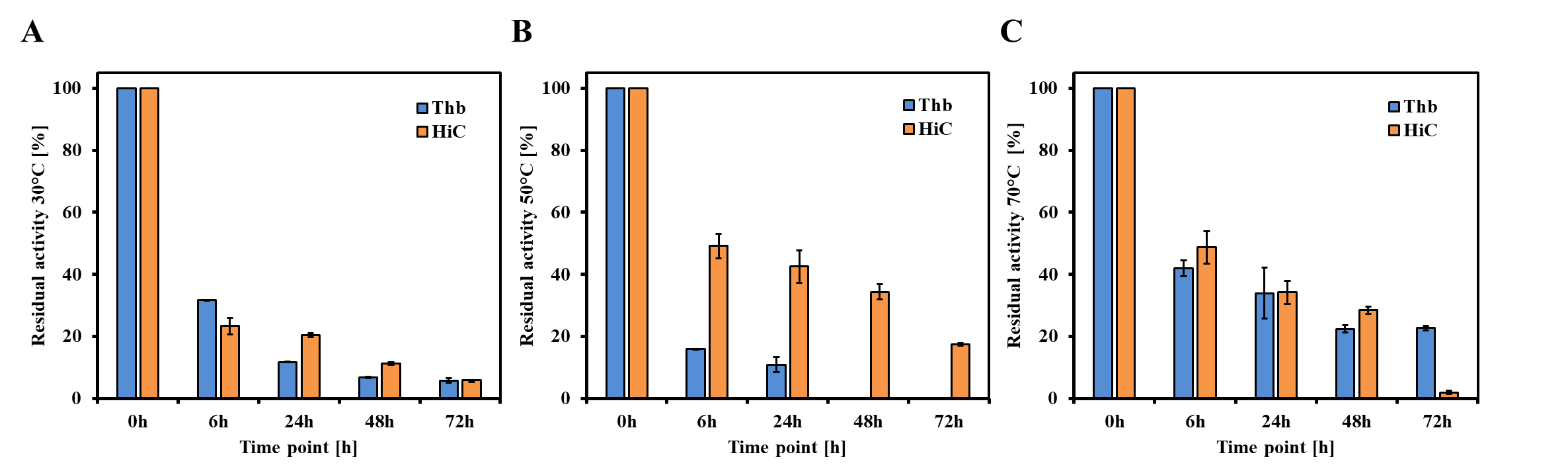


**Figure S4**. Long-term stability of Hic and Thb at 1 M KPO pH 8 measured on *p-*NPB after 0 h, 6 h, 24 h, 48 h, 72 h at different temperatures. A: residual activity [%] at 30°C; B: residual activity [%] at 50°C; C: residual activity [%] at 70°C. ,


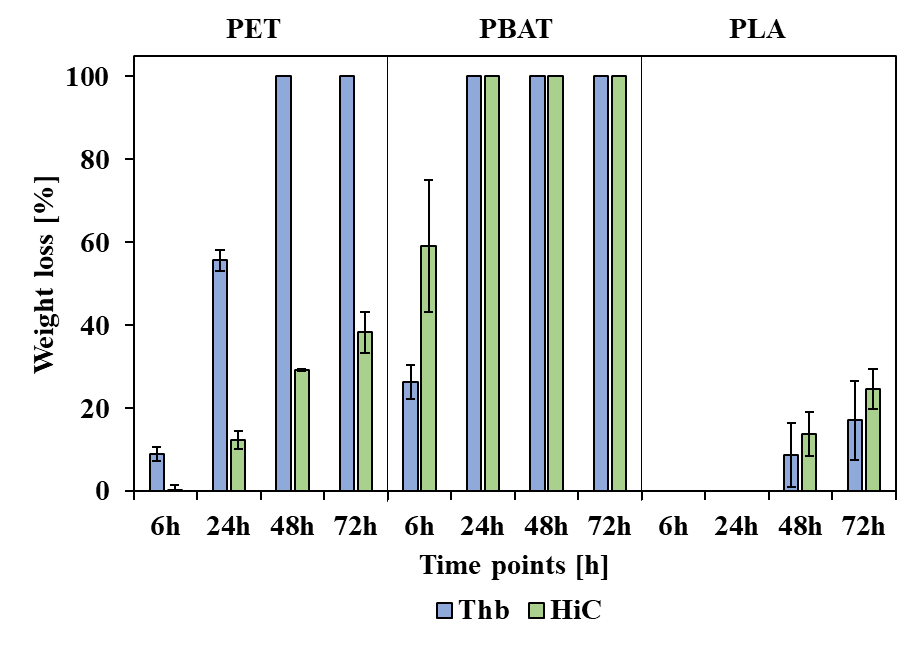


**Figure S5**. Weight loss of PET, PBAT and PLA upon incubation with Thb and HiC in presence of 2 mM CaCl_2_. Results are an average of triplicate measurements. Light blue bars: Thb, light green bars: HiC.


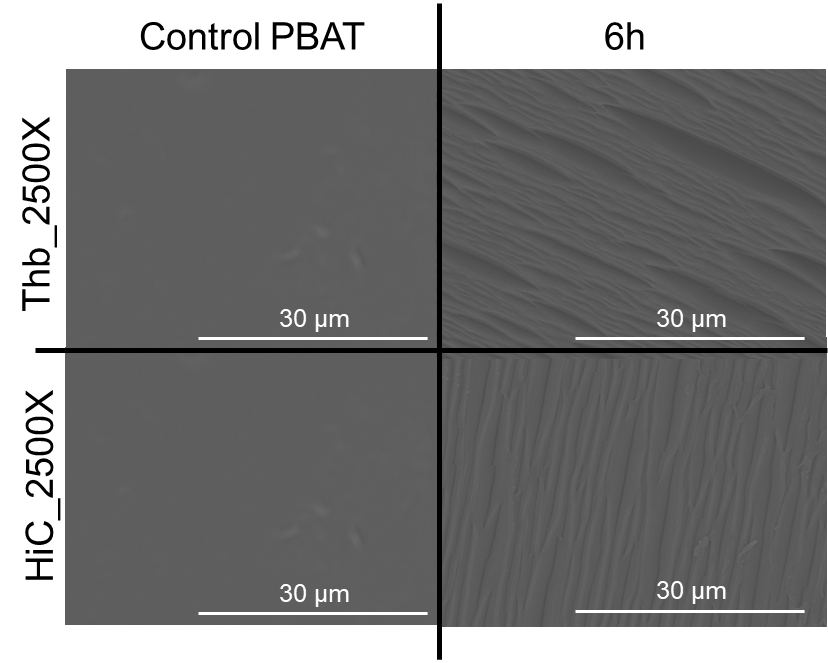


**Figure S6**. SEM at 2500X of PBAT treated with HiC or Thb at 6-hour time point


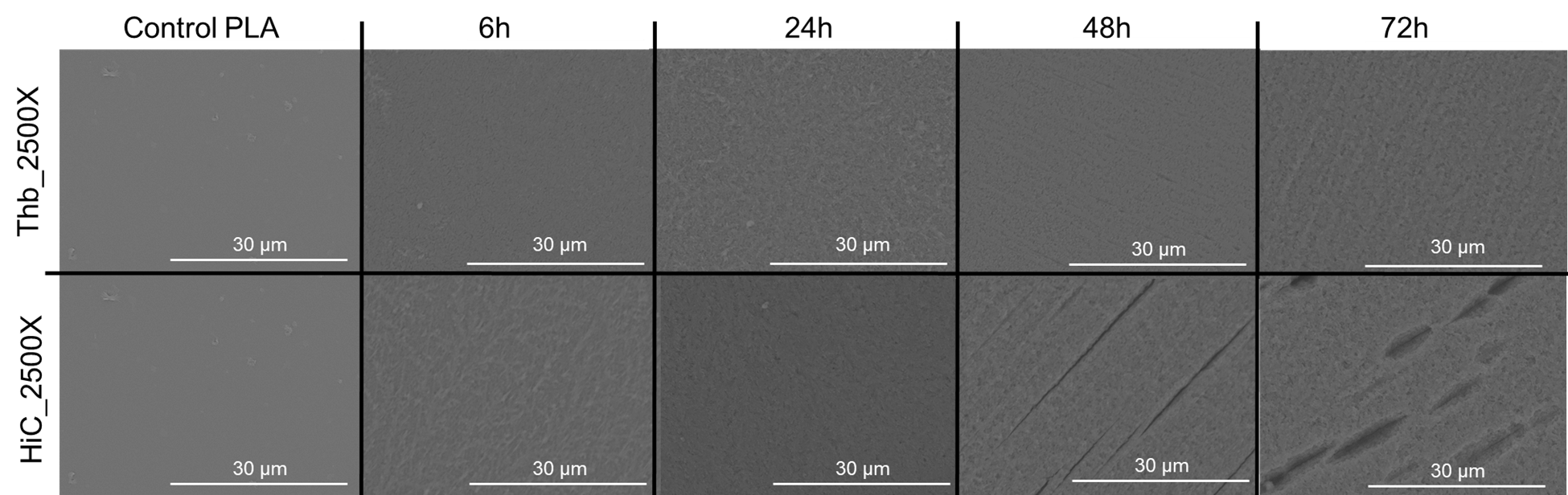


**Figure S7.** SEM at 2500X of PLA from HiC and Thb in presence of CaCl_2._


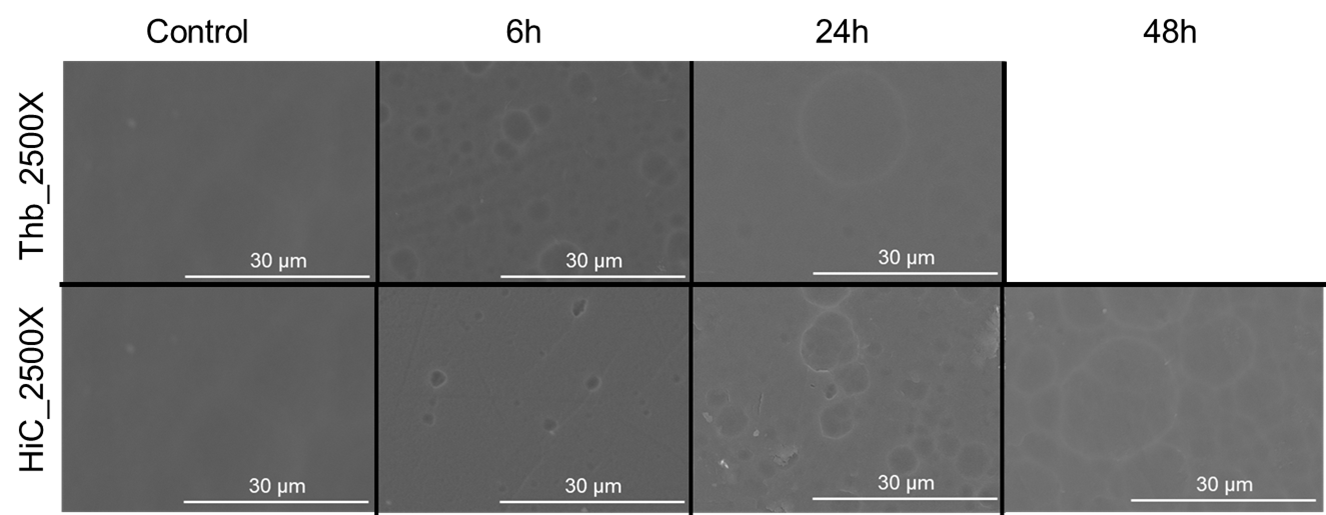


**Figure S8**. SEM at 2500X of PET from HiC and Thb


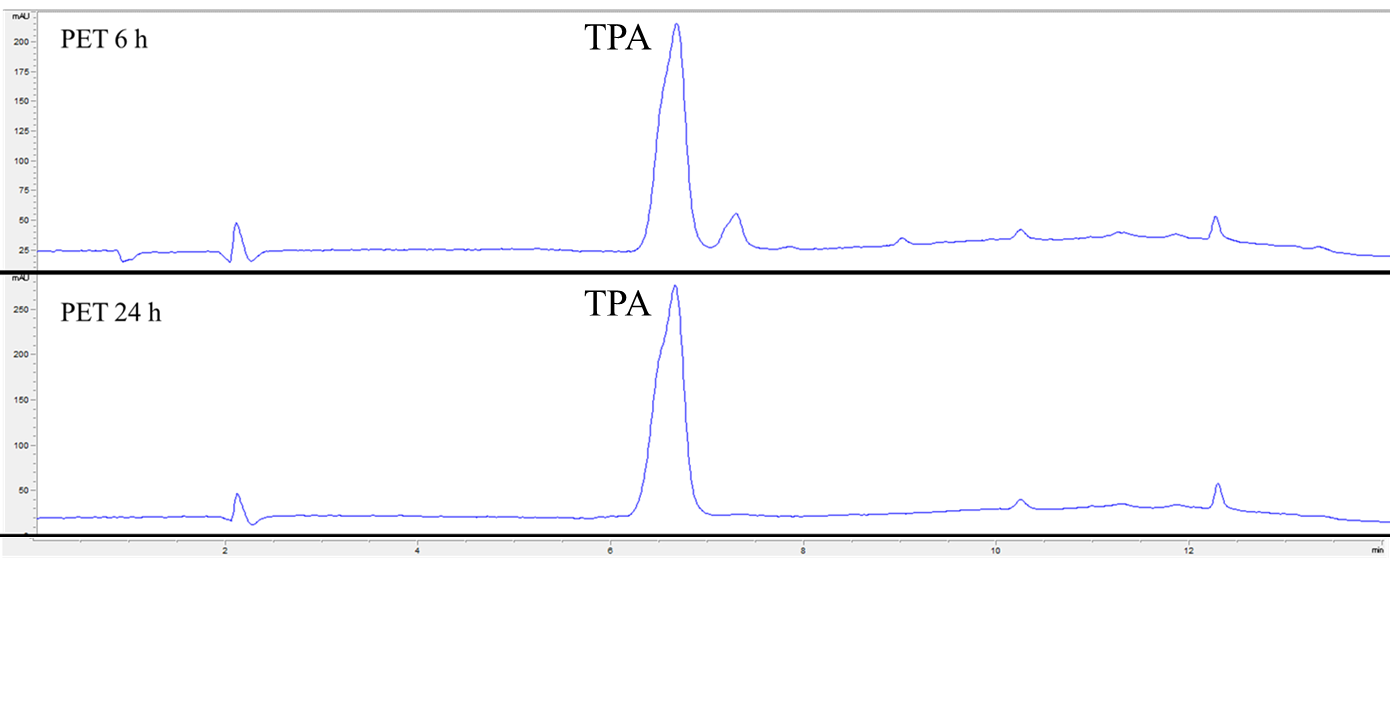


**Figure S9.** Stacked chromatograms of PET time points as measures *via* UV-HPLC. The time points are indicated in the figure (6 h, 24 h, 48 h, 72 h). Retention time TPA: 6.4 minutes.


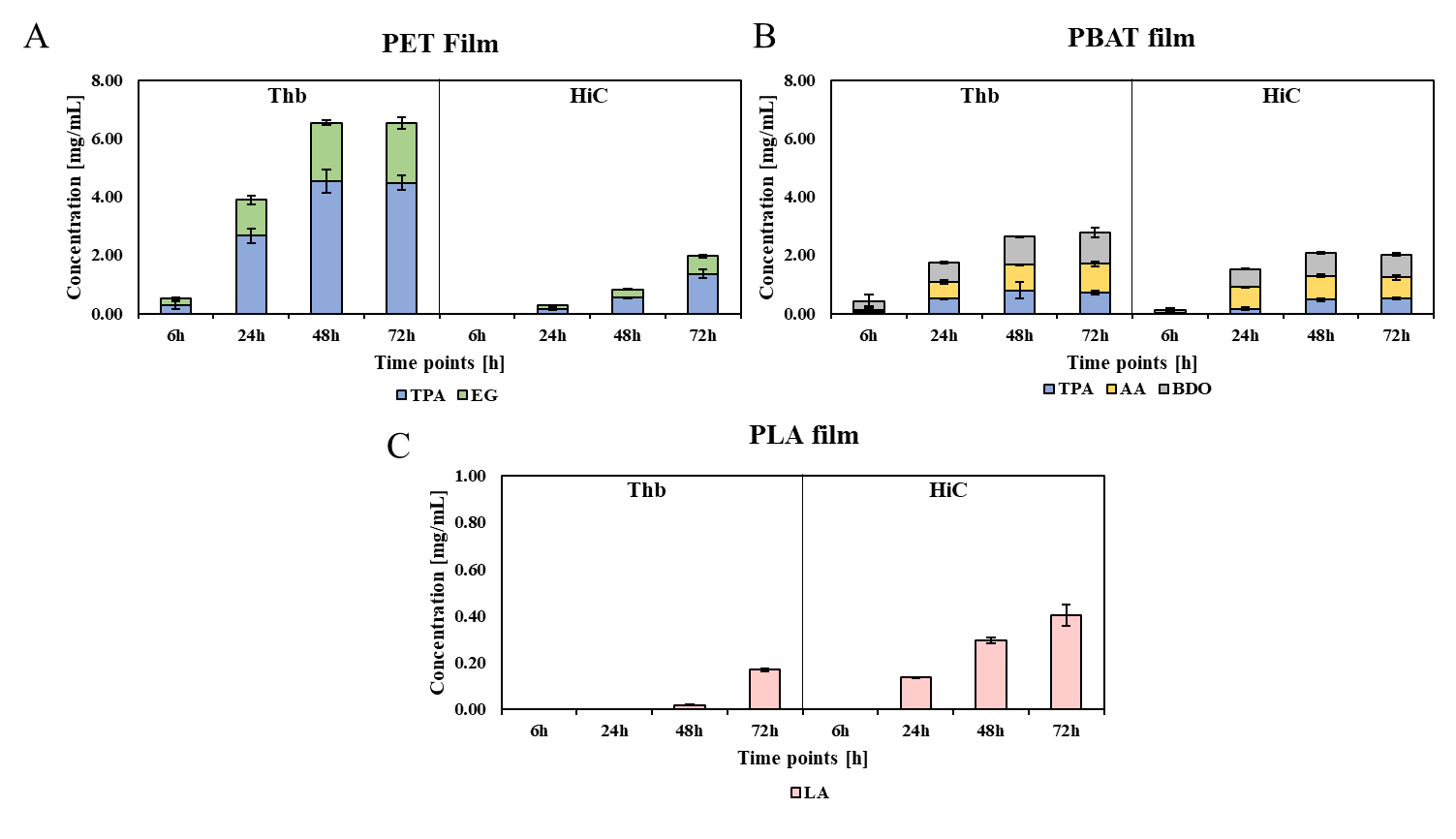


**Figure S10.** Monomers (mg/mL) released by PET, PBAT and PLA in films incubated with 1µM HiC or Thb. Blue bars: TPA; green bars: EG. Pink bars: LA; light green bars: SA; grey bars: BDO; yellow bars: AA. A: PET film, B: PBAT film, C: PLA film.


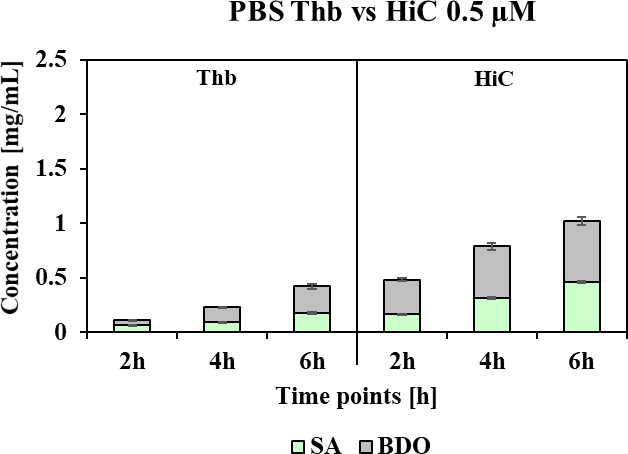


**Figure S11**. Soluble monomers traced in each time point PBS hydrolysate at 0.5 µM Thb vs HiC. Values were normalized by the initial weight of the polymer in mg. Light green bars: SA; grey bars: BDO.


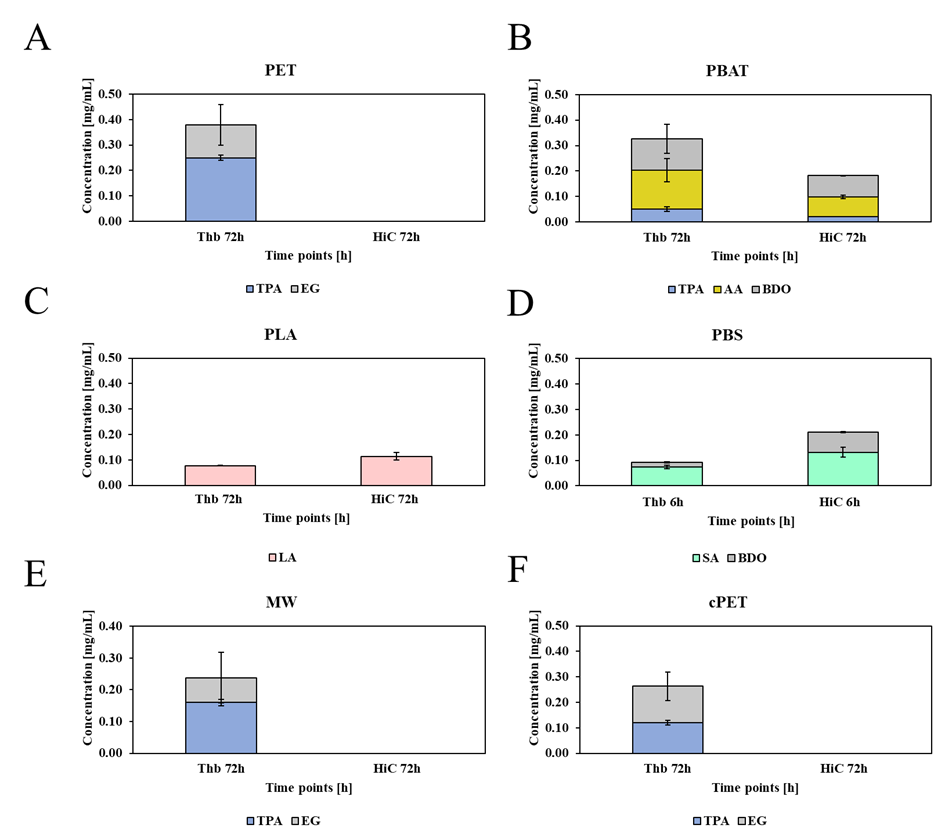


**Figure S12.** Soluble monomers released from PET, PBAT, PLA, PBS, Mixed waste and crystalline PET hydrolysate by the enzymes Thb and HiC incubated at 30°C for 72 h. Values were normalized by the initial weight of the polymer in mg. Blue bars: TPA; green bars: ethylene glycol (EG). Pink bars: lactic acid (LA); light green bars: SA; grey bars: 1,4-butanediol (BDO); yellow bars: adipic acid (AA). A: PET; B: PBAT; C: PLA; D: PBS; E: MW; F: crystalline PET.


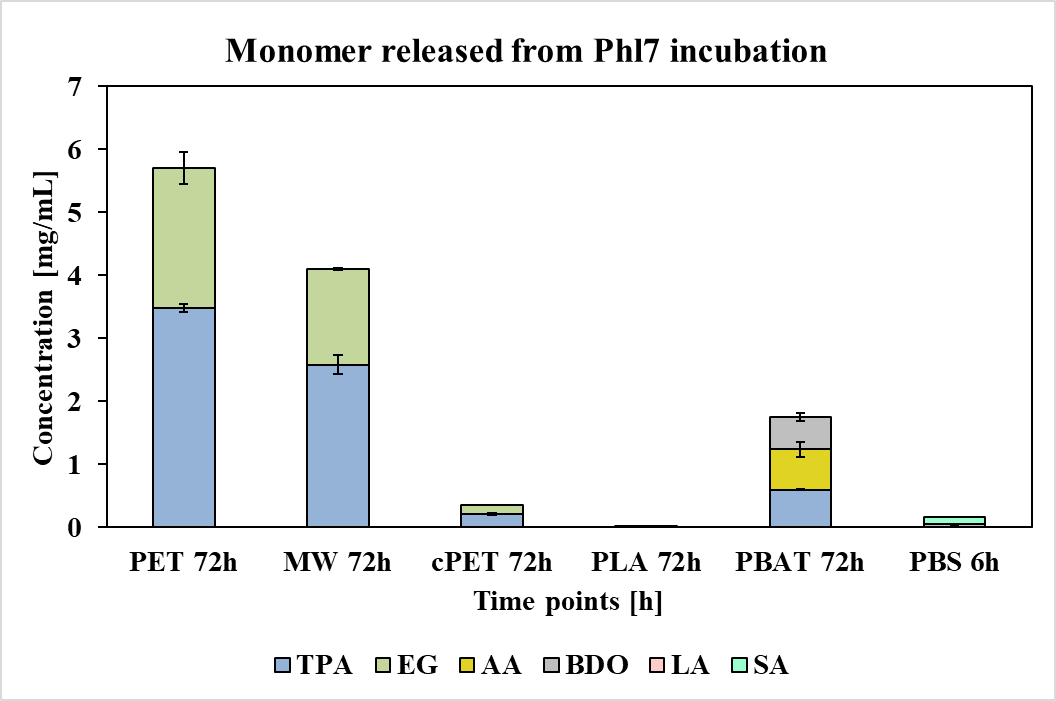


**Figure S13.** Soluble monomers released from PET, PBAT, PLA, PBS, Mixed waste and crystalline PET hydrolysates by the enzyme Phl7 incubated at 70°C for 72 h.


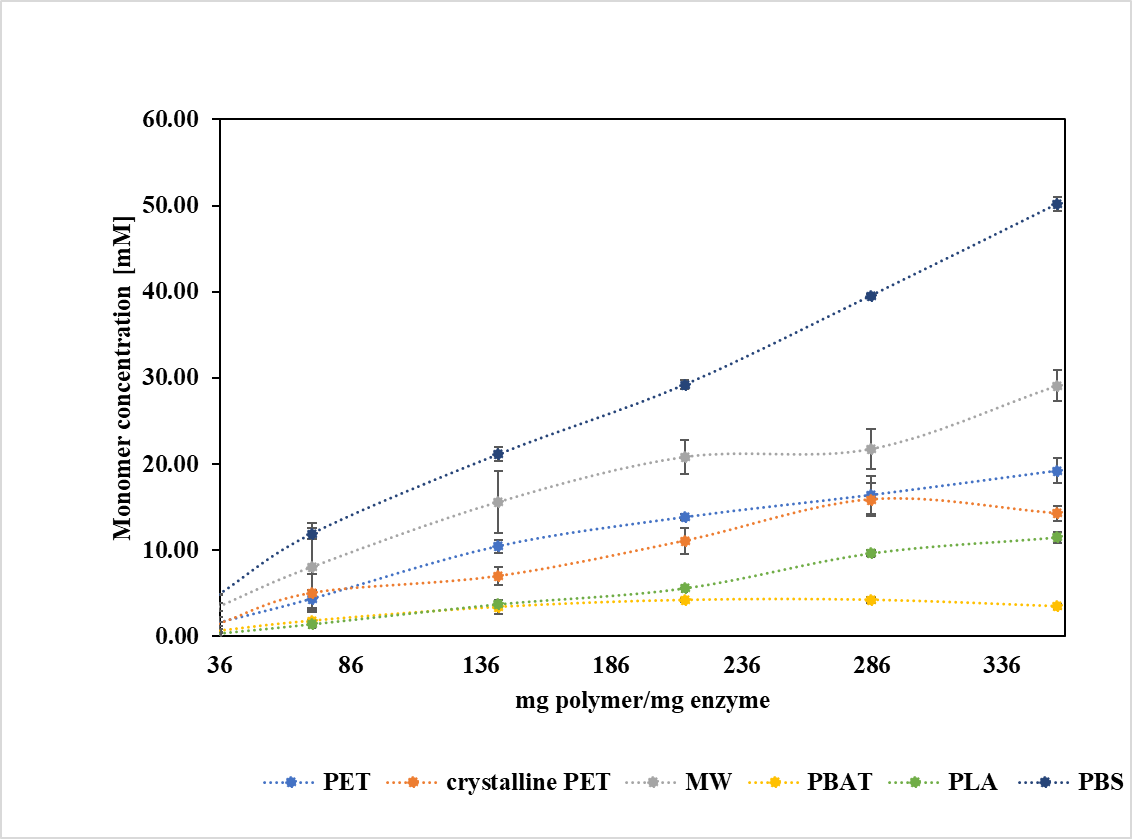


**Figure S14.** Monomer concentration trend dependent on the ratio mg polymer/mg enzyme. Terephthalic acid (TPA) was plotted as monomer for PET (blue), crystalline PET (orange), MW (grey), PBAT (yellow); lactic acid (LA) was plotted for PLA (green line) and succinic acid (SA) was plotted for PBS (dark blue).


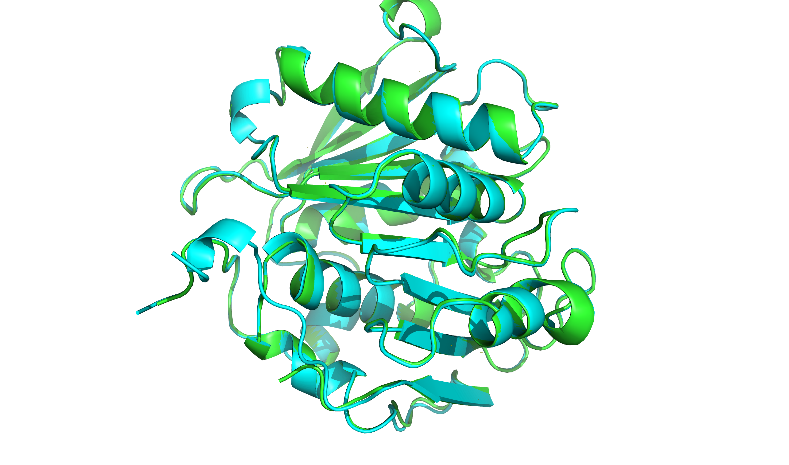


**Figure S15**. Comparison of the two models: homology (green) vs Alphafolds 3 (cyan). The root-mean standard deviation after alignment is 0.16 Å.
